# *Helicobacter pylori* Eradication Therapy and the Risk of Colorectal Cancer: A Population‐Based Nationwide Cohort Study in Sweden

**DOI:** 10.1111/hel.70001

**Published:** 2024-11-20

**Authors:** Qing Liu, Omid Sadr‐Azodi, Lars Engstrand, Katja Fall, Nele Brusselaers

**Affiliations:** ^1^ Centre for Translational Microbiome Research, Department of Microbiology, Tumor and Cell Biology Karolinska Institutet Stockholm Sweden; ^2^ Department of Clinical Sciences, Intervention and Technology Karolinska Institutet Stockholm Sweden; ^3^ Department of Surgery Capio Saint Göran Hospital Stockholm Sweden; ^4^ Clinical Epidemiology and Biostatistics School of Medical Sciences Örebro University Örebro Sweden; ^5^ Institute of Environmental Medicine Karolinska Institutet Stockholm Sweden; ^6^ Global Health Institute University of Antwerp Antwerp Belgium; ^7^ Department of Public Health and Primary Care Ghent University Ghent Belgium; ^8^ Department of Women's and Children's Health Karolinska Institutet Stockholm Sweden

**Keywords:** antibiotics, cancer risk, colorectal adenocarcinoma, *Helicobacter pylori* eradication therapy, proton pump inhibitors

## Abstract

**Background:**

*Helicobacter pylori* ( *H. pylori*) is an established gastric carcinogen, also associated with an increased risk of colorectal cancer. Therefore, we suspected that *H. pylori* eradication lowers the risk of colorectal cancer.

**Material and Methods:**

We assessed if *H. pylori* eradication therapy is associated with a reduced risk of colorectal adenocarcinoma in a population‐based nationwide cohort study. This study included all Swedish adults with at least one recorded *H. pylori* eradication episode between July 2005 and December 2012, based on the high‐quality Swedish health registries. Colorectal adenocarcinoma risks were compared to the Swedish background population, presented as standardized incidence ratios (SIRs) and 95% confidence intervals (CIs), accounting for age, sex, calendar period, tumor location (left or right sided), stage, and number of eradication episodes, from 1 year after eradication and onward.

**Results:**

Among 80,381 individuals receiving *H. pylori* eradication therapy (average follow‐up 4.1 years), 282 were diagnosed with colorectal cancer (97.2% adenocarcinoma). Overall, *H. pylori* eradication was associated with an elevated risk of colorectal adenocarcinoma (SIR 1.27, 95% CI: 1.12–1.43). The colorectal adenocarcinoma risk was increased 1–2 years after eradication (SIR 1.42, 95% CI: 1.17–1.72), then decreased 2–4 years (SIR 0.80, 95% CI: 0.65–0.98) and 4–6 years (SIR 0.76, 95% CI: 0.57–0.99), yet not ≥ 6 years (SIR 1.36, 95% CI: 0.78–2.21) after eradication compared to the general population. Overall, right‐sided (SIR 1.47, 95% CI: 1.21–1.76) and left‐sided (SIR 1.35, 95% CI: 1.09–1.67) colon adenocarcinomas risks were higher among eradicated individuals than the general population.

**Conclusion:**

*H. pylori* eradication was not associated with a clear and consistent reduction of colorectal cancer in our Swedish cohort.

## 1 Introduction

Colorectal cancer is the third most prevalent cancer worldwide and the fourth major cause of cancer‐related death, with an increasing incidence among younger adults and particularly high incidences in Australia/New Zealand and European regions [1].

Increasing epidemiological evidence since the early 1990s, has suggested that *H. pylori* infection increases the risk of colorectal cancer [2, 3, 4, 5, 6], and *H. pylori* has been detected in colorectal lesions [7, 8, 9]. *H. pylori* infection is associated with an elevated risk of colorectal polyps, colorectal neoplasms, and colorectal cancer, with recent meta‐analyses reporting pooled odds ratios between 1.27 and 1.86 for the colorectal cancer risk in individuals with *H. pylori* ‐infection compared to those without [2, 3, 4, 5]. The exact mechanism by which *H. pylori* contributes to colorectal cancer is still not fully understood as it seems not to thrive and survive in the lower gastrointestinal tract [6]. Yet, this bacterium is believed to reduce gastric acidity, consequently leading to gut microbiota changes in the stomach and the lower gastrointestinal tract, contributing to chronic inflammation, systemic inflammatory immune responses, and a cancer‐promoting environment [6, 10, 11]. Some research suggest that *H. pylori* is more frequently detected in right‐sided colorectal cancer, than left‐sided colorectal cancer [9]. Right‐sided and left‐sided colorectal cancer do seem to have other risk and prognostic factors, including differences in fecal exposure, and different clinical symptoms and time to diagnosis [12, 13].

If *H. pylori* is truly associated with an increased risk of colorectal cancer, this could imply that the eradication of *H. pylori* reduces the risk of colorectal cancer by reversing *H. pylori* ‐induced effects, as also suggested by meta‐analyses for gastric cancer [14, 15, 16, 17].

Our previous studies in Sweden concluded that *H. pylori* eradication, generally consisting of a 7‐day course of proton‐pump inhibitors (PPI) and two antibiotics [18], appeared to contribute to a reduced risk of gastric cancer over time since eradication [19, 20]. As gastric reflux is a major risk factor for esophageal cancer, and *H. pylori* seems to reduce gastric acidity, it was postulated that the risk of esophageal cancer could increase after eradication. Yet, we did not find an increase in Barrett's esophagus or esophageal cancer with time after eradication [19, 20]. It remains unclear how *H. pylori* eradication affects colorectal cancer risk, as only few Southern Asian studies investigated this association (suggesting a gradual decreasing risk) [21, 22]. Although gastric cancer incidence remains high in several Asian regions, the incidence of colorectal cancer is globally among the lowest, an opposite situation compared to Europe, which makes the role of *H. pylori* and its eradication even more interesting [1, 23, 24, 25].

Although *H. pylori* eradication is only based on a short exposure period to antibiotics and PPIs, there is increasing evidence that both components of the eradication therapy, PPIs and antibiotics (most commonly clarithromycin combined with amoxicillin or metronidazole), may negatively affect gastrointestinal carcinogenesis, if used over prolonged periods of time: PPI‐induced reduction in acid secretion may foster intestinal bacterial overgrowth and microbiome alterations [26], and some association studies have linked PPI use to increased risk of colorectal cancer besides the large amount of studies linking PPIs with an increased risk of gastric cancer [26, 27, 28]. Two recent meta‐analyses also associated antibiotic use overall, and most subclasses, with an increased risk of colorectal cancer [29, 30, 31]. Of the broader antibiotic classes most commonly used for *H. pylori* eradication, nitroimidazoles/metronidazole showed the largest pooled increase of colorectal cancer (risk ratio = 1.28), followed by penicillins (RR = 1.16) and macrolides (RR = 1.04) [29].

The aim of this study was to assess the association between *H. pylori* eradication and the risk of colorectal cancer in a European setting with low *H. pylori* incidence, compared to the general background population of the same age and sex, accounting for the therapy episode, duration of follow‐up, different anatomical locations and tumor stages.

## 2 Materials and Methods

### 2.1 Study Design

**FIGURE 1:**
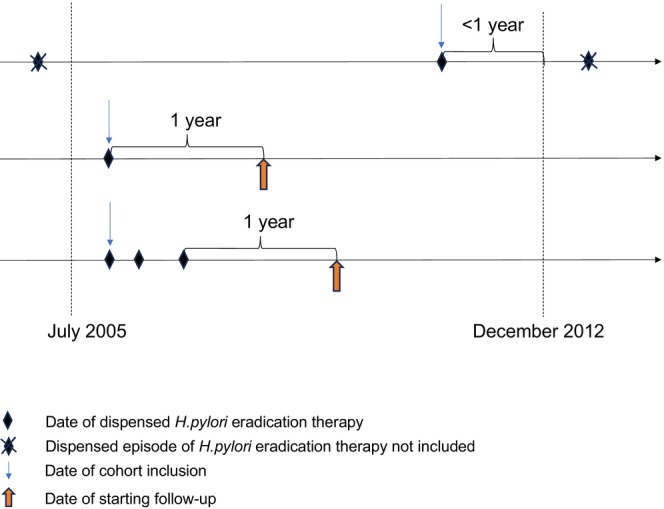
Timeline examples showing date of *Helicobacter pylori* ( *H. pylori* ) eradication treatment(s), date of cohort inclusion, and date of starting follow‐up during 2005–2012.

This is a nationwide population‐based cohort study based on the Swedish Prescribed Drug Registry, Cancer Registry, and Causes of Death Registry, and the total population registry (16–18). This cohort included all adults (age ≥ 18 at the last dispensed *H. pylori* eradication prescription) without any pre‐diagnosed malignancies who received ≥ 1 episode of *H. pylori* eradication therapy between July 1, 2005 (start of the Prescribed Drug Registry [32]), and December 31, 2012 (Figure S1) [18, 19, 20]. Individuals were followed up from 1 year after the last eradication date (to minimize detection and lag time bias) until the occurrence of colorectal cancer, death, or the end of the study period (Figure 1). The identified users of *H. pylori* eradication were compared to the general population of the same age, sex, and calendar period. The Regional Ethical Review Board in Stockholm approved the study (2014/1291–31/4), and informed consent was not required. The study is reported according to the Strengthening the Reporting of Observational Studies in Epidemiology (STROBE) statement [33].

### 2.2 Exposure

Exposure was defined as ≥ 1 dispensed *H. pylori* eradication prescription defined as a standard package or a recommended combination regimen in Sweden [18]. The standard package currently in use for *H. pylori* eradication consists of combined regimens with esomeprazole, amoxicillin, and clarithromycin for 7 days (Anatomical Therapeutic Chemical Classification [ATC] code, A02BD06). Recommended combination regimens can also be dispensed separately of a proton pump inhibitor (ATC code, A02BC) together with two of these antibiotics: amoxicillin (ATC code, J01CA04), clarithromycin (ATC code, J01FA09), and metronidazole (ATC code, J01XD01); for all combination regimens, the antibiotics had to be dispensed on the exact same date, whereas the PPI was allowed to be dispensed within a window of 60 days before or 5 days after the antibiotic prescription as some individuals may be maintenance users of PPIs and do not necessarily dispense another PPI prescription on the same day as the antibiotic prescriptions [14]. In this study, we focused on the standard package and the above‐described combination regimens, which accounted for 95.4% of the identified eradication regimens in Sweden [5]. According to a recent meta‐analysis, this first‐line treatment efficacy is around 95% in Europe [34]. Other eradication episodes only using alternative combination regimens for *H. pylori* eradication therapy (other antibiotic combinations particularly for non‐primary eradication episodes), were excluded from the analysis, meaning the standard eradication was used to determine start of follow‐up.

### 2.3 Outcomes

The primary outcome was defined as the first diagnosis of colorectal adenocarcinoma by the International Classification of Diseases, 10th edition (ICD‐10 code for colorectal cancer: C19‐C21), combined with the 096 histological code for adenocarcinoma, which accounts for > 95% of all colorectal cancer cases [35]. Right‐sided colon, left‐sided colon, and rectum adenocarcinoma were separated by their first diagnosed registry records from the unspecified or overlapping tumor locations; and anal cancer was calculated for comparison reasons (codes in Table S1). Colorectal adenocarcinoma was staged using the tumor‐node‐metastasis classification and Staging of Colon Cancer (American Joint Committee on Cancer 8th edition): stage 0‐I (Tis, N0, M0 for stage 0; T1‐2, N0, M0 for stage I), II (T3‐4, N0, M0), III (T1‐4, N1‐2, M0), and IV (any T, any N, M1) [36]. The different anatomical locations and stages of colorectal adenocarcinoma were used in the subgroup analysis.

### 2.4 Confounders

Age at the last dispensed prescription (< 60, 60–69, and ≥ 70 years old), sex (men and women), and calendar period at the year of the last eradication treatment of *H. pylori* (2005–2006, 2007–2009, and 2010–2012) were categorized in the analysis of colorectal adenocarcinoma. The number of *H. pylori* eradication episodes received was classified as 1 and ≥ 2 to identify recurrent or refractory cases. A single episode of *H. pylori* eradication therapy was considered as a course of successful *H. pylori* eradication dispensation, while multiple episodes most likely reflected failure of eradication and follow‐up time was calculated from the last episode (+1 year lag time). The period of follow‐up was classified as 1–2 years, 2–4 years, 4–6 years, and ≥ 6 years.

### 2.5 Statistical Analyses

Standardized incidence ratios (SIRs) with 95% CIs were calculated for the risk of colorectal adenocarcinomas after *H. pylori* eradication therapy comparing with the entire Swedish population during the same observation period, standardizing for age, sex, and calendar period. Standardization was performed according to Breslow and Day's method and the allocation of person‐years followed the Clayton's algorithm [37]. Using this method, we compared the risk of colorectal cancer between those exposed to *H. pylori* eradication and the full Swedish background population of the same sex (men or women), age group (categorized as 18–39, 40–49, 50–59, 60–69, or ≥ 70 years), and calendar period. SIRs were calculated by dividing the observed number of cancer cases with the expected number, therefore accounting for changes in calendar period and aging during the study period. Subgroup analyses of colorectal adenocarcinoma were conducted for the number of eradication therapy episodes (i.e., 1 and ≥ 2), duration of follow‐up (i.e., 1–2 years, 2–4 years, 4–6 years, and ≥ 6 years), tumor locations (i.e., right‐sided colon, left‐sided colon, and rectum), and tumor stages (i.e., Stage 0/I, II, III, and IV) of colorectal adenocarcinoma. We used STATA 14.2 (RRID: SCR_012763, Stata Corp, Texas, USA) for the analyses.

## 3 Results

### 3.1 Cohort Characteristics

This cohort included 80,381 individuals who were followed up for at least a year after receiving *H. pylori* eradication therapy during the study period (Table 1). The average duration of follow‐up was 4.1 years. By the time of the last dispensed *H. pylori* eradication therapy, most of the exposed population (60.1%) was under the age of 60, and women (53.7%) outnumbered men (46.3%). During the observation period, 92.1% (*N* = 74,011) of the exposed population received only one episode of *H. pylori* eradication therapy (Table 1). Out of 282 primary colorectal cancer cases, 274 developed colorectal adenocarcinoma (97.2%), the majority of which were identified as stage II (*N* = 59) and stage III (*N* = 67) (Table 1). Colorectal adenocarcinomas were categorized as right‐sided (*N* = 112), left‐sided (*N* = 88), and rectum adenocarcinoma (*N* = 71), with the remaining locations classified as overlapping or unspecified (*N* = 3) (Table 2).

**TABLE 1:** Descriptive table of all individuals received *H. pylori* eradication therapy during 2005–2012.

Characteristics of exposed population	Exposed population	
	Number	Percentage (%)
Total	80,381	100.0
Sex		
Men	37,190	46.3
Women	43,191	53.7
Age at follow‐up, years		
18–59	48,277	60.1
60–69	15,387	19.1
≥ 70	16,717	20.8
Calendar period		
2005–2006	20,565	25.6
2007–2009	37,276	46.4
2010–2012	22,540	28.0
Follow‐up years (median)	4.1	—
Number of *H. pylori* eradication therapy episodes		
1	74,011	92.1
≥ 2	6370	7.9

**TABLE 2:** Risk of colorectal adenocarcinoma in different sex, age, calendar period, numbers of *H. pylori* eradication therapies, tumor locations and stages between 2005 and 2012, expressed by standardized incidence rate ratio (SIR) and confidence interval (CI).

	Colorectal adenocarcinoma	
	Number	SIR (95% CI)
Total colorectal adenocarcinoma	274	1.27 (1.12–1.43)
Sex		
Men	142	1.21 (1.02–1.42)
Women	132	1.34 (1.12–1.59)
Age at follow‐up, years		
< 60	42	0.90 (0.58–1.33)
60–69	77	1.16 (0.89–1.48)
≥ 70	155	1.39 (1.20–1.61)
Calendar period		
2007–2009	88	1.41 (1.13–1.73)
2010–2012	186	1.22 (1.05–1.41)
Numbers of *H. pylori* eradication therapy episodes		
1	258	1.27 (1.12–1.44)
≥ 2	16	1.21 (0.69–1.97)
Tumor locations		
Right‐sided colon adenocarcinoma	112	1.47 (1.21–1.76)
Left‐sided colon adenocarcinoma	88	1.35 (1.09–1.67)
Rectal adenocarcinoma	71	0.94 (0.74–1.19)
Unspecified colorectal adenocarcinoma	3	—
Anal cancer	7	1.29 (0.52–2.66)
Stages of colorectal adenocarcinoma		
Stage 0/I	42	1.38 (0.99–1.86)
Stage II	59	1.10 (0.84–1.42)
Stage III	67	1.28 (0.99–1.62)
Stage IV	56	1.26 (0.96–1.64)
Missing	50	1.40 (1.04–1.85)

### 3.2 Risk of Colorectal Cancer and Adenocarcinoma

Compared to the general population, individuals who received *H. pylori* eradication had a higher overall risk of colorectal adenocarcinoma (SIR = 1.27, 95% CI: 1.12–1.43) (Table 2). Both women (SIR = 1.34, 95% CI: 1.12–1.59) and men (SIR = 1.21, 95% CI: 1.02–1.42) presented with a modestly elevated risk of developing colorectal adenocarcinoma following *H. pylori* eradication therapy when compared to the general population. Among age groups, people aged over 70 years had a notably higher risk of colorectal adenocarcinoma (SIR = 1.39, 95% CI: 1.20–1.61) than the general population of the same age, while no significant differences were identified in other age groups. Moreover, those who received a single *H. pylori* eradication treatment showed a 27% increased risk of colorectal adenocarcinoma compared to the general population (SIR = 1.27, 95% CI: 1.12–1.44), while no significant association was found for multiple eradication episodes (SIR = 1.21, 95% CI: 0.69–1.97) (Table 2).

### 3.3 Tumor Location and Stage

For individuals who had eradication therapies, the risk of right‐sided (SIR = 1.47, 95% CI: 1.21–1.76) and left‐sided (SIR = 1.35, 95% CI: 1.09–1.67) colon adenocarcinoma were both higher than in general population, whereas the association with rectal adenocarcinoma was not significant (SIR = 0.94, 95% CI: 0.74–1.19). No association was found for anal cancer (SIR = 1.29, 95% CI: 0.52–2.66). None of the sub‐analyses for tumor stage reached statistical significance, with the effect estimates ranging between SIR = 1.10 (stage II) and SIR = 1.38 (stage 0/I) (Table 2).

### 3.4 Duration of Follow‐Up

During the first follow‐up period (1–2 years after eradication), a higher risk of colorectal adenocarcinoma (SIR = 1.42, 95% CI: 1.17–1.72) was observed compared to the general population, particularly for right‐sided colon adenocarcinoma (SIR = 1.60, 95% CI: 1.15–2.16) (Table 3). During the second and third follow‐up period, the risk of colorectal adenocarcinoma was significantly lower than the background population with SIR = 0.80, 95% CI: 0.65–0.98 (2–4 years after eradication) and SIR = 0.76, 95% CI: 0.57–0.99 (4–6 years after eradication) (Table 3). The risk was again higher, although not statistically significant, compared with the background population for the last follow‐up period (≥ 6 years) with SIR = 1.36 (0.78–2.21). The subgroup analyses per anatomical location showed similar results, yet did not reach statistical significance.

**TABLE 3:** Risk of colorectal adenocarcinoma by anatomical location and duration of follow‐up, expressed by standardized incidence rate ratios (SIR) and confidence intervals (CI).

	Colorectal adenocarcinoma		Right‐sided colon adenocarcinoma		Left‐sided colon adenocarcinoma		Rectal adenocarcinoma	
	Number	SIR (95% CI)	Number	SIR (95% CI)	Number	SIR (95% CI)	Number	SIR (95% CI)
Total	274	1.27 (1.12–1.43)	112	1.47 (1.21–1.76)	88	1.35 (1.09–1.67)	71	0.94 (0.74–1.19)
Duration of follow‐up, years								
1–2	105	1.42 (1.17–1.72)	42	1.60 (1.15–2.16)	33	1.49 (1.02–2.09)	28	1.10 (0.73–1.60)
2–4	99	0.80 (0.65–0.98)	42	0.96 (0.69–1.30)	34	0.92 (0.64–1.28)	22	0.52 (0.32–0.78)
4–6	54	0.76 (0.57–0.99)	23	0.89 (0.56–1.33)	16	0.75 (0.43–1.22)	15	0.62 (0.35–1.03)
≥ 6	16	1.36 (0.78–2.21)	5	1.19 (0.38–2.78)	5	1.42 (0.46–3.31)	6	1.48 (0.54–3.22)

## 4 Discussion

The results of this Swedish nationwide cohort study did not provide evidence for a clearly decreasing risk of colorectal adenocarcinoma after *H. pylori* eradication treatment. Although the risk was reduced 3–6 years after eradication therapy, it was unclear beyond 6 years. The observed initial excess risk could potentially be attributed to residual effects of *H. pylori* . Alternatively, it may be explained by increased detection of yet undiagnosed cancers among patients receiving medical attention in connection with their eradication therapy (detection bias—a mechanism that could also explain a compensatory reduction in risk during the following few years).

Although exploring underlying pathways is beyond the scope of this epidemiological project, our project may help in determining if potential mechanisms actually may have an effect on population level. The evidence regarding long‐term safety of any drug class regarding carcinogenicity remains limited, as it is challenging to collect the required large‐scale population data and have sufficiently long‐follow‐up, combined with detailed clinical and demographic data to adjust for confounding effects. For this present project, we did focus on a short‐term exposure and the hypothesis is that it is the presence of *H. pylori* affecting the cancer risk, and not the treatment itself. As described in the introduction, the treatment components itself, PPIs and antibiotics, may also affect cancer risk, yet presumably longer exposure times (and higher accumulated dosages) would be required to affect carcinogenesis detectable on population‐level, and differences by sex, age, and other patient characteristics are likely, and other confounding factors should be accounted for. As such, the reports of PPIs may be more worrisome, as a large group of users become maintenance users, using PPIs for multiple years, while antibiotics are usually administered over a short period of time. Also taking into account recent advances in the microbiome field, we do believe prescribed drug use should not be overlooked as a potential risk factor, or confounder, when addressing carcinogenesis. Our study has several strengths including the large cohort size, and population‐based design, based on the large and unique Swedish Health registries with generally high validity [32, 38, 39, 40]. The Swedish Prescribed Drug Registry is virtually complete for the entire Swedish population and includes all prescribed outpatient drugs, with patient‐identifiable data missing in < 0.3% of all items [32, 39]. The Swedish Cancer Registry has a coverage of over 96% of all cancer diagnoses [41]. The risk of selection bias or misclassification by exposure was limited—as *H. pylori* eradication is only available on prescription, and usually administered in outpatient setting [18]. We only included the most common combination of PPI and antibiotics; which covers around 95% of all *H. pylori* as described in our previous work [18]. Unfortunately, we do not have information on seroprevalence of *H. pylori* , at any time prior or after eradication—as this is not routinely collected in the Swedish Patient Registry (and coverage of these diagnosis is low as seen in our previous studies) [42]; yet 8% of the cohort received multiple eradication episodes indicating the initial eradication was not successful. During the study period, there was no nationwide colorectal cancer screening program rolled out, so screening participation should not have had a major effect on the (timing of) diagnosis [43, 44].

Using standardization for estimation risks implies calculation of expected risks compared to observed risk based on generally available risk estimates of the general Swedish population of the same sex, age, and calendar period. This method does not allow for additional adjustment for other potential risk factors including family history and socioeconomic status, information we did not have on the entire background population, and/or that was not recorded in the nationwide health registries (diet, smoking, and body mass index). We also based our study on the nationwide Cancer Registry as this project was part of a larger data collection looking at different drug classes and cancer types. There is now a valid and more detailed colorectal cancer quality registry (established for rectal cancer in 1995, and colon cancer since 2007). Quality registers do not have mandatory registration; yet this registry obtains a coverage of 98.5% for colon cancer [45]. This resource could be explored in the future, especially when follow‐up after eradication will be longer, as we suspect our follow‐up of max 7.5 years after eradication was still too short to have reliable and complete long‐term results for colorectal cancer [29, 46]. As a test‐and‐treat approach is followed in Sweden, there is no general screening for *H. pylori* in asymptomatic individuals, and all individuals with detected *H. pylori* should have received (or been offered) eradication, as also recommended by clinical guidelines [47]. Therefore, there is no control group with proven *H. pylori* presence not receiving eradication, which complicates disentangling the effects of the bacterium and the therapy. A recent large study from the United States, based on the Veterans database, did compare individuals with detected *H. pylori* not receiving treatment with those receiving eradication, which did suggest lower risks in those receiving eradication (with a follow‐up of up to 20 years) [48].

Our findings for the 1–2 years after eradication align with the results from previous meta‐analyses on *H. pylori* infection [2, 3, 4, 5], as too early after eradication the effect of eradication on cancer risk may not yet be visible as the risk remains higher in the eradicated group compared to the general population. In the subsequent follow‐up intervals, the risk decreased below that of the general population but due to small numbers, the estimates beyond 6 years were unclear. Important to highlight is the so‐called “chronology of cancer,” a concept used to “describe the nature of cancer through the measure of time.” [46]. Carcinogenesis is considered a multistep process, from initiation to promotion and progression, often estimated to take around 10 years from a premalignant polyp to colorectal cancer [46]. If eradication occurs after this carcinogenic process has already been initiated, it may still slow down progression, but may not entirely eliminate the risk. As colorectal cancer is a relatively slow progressing cancer (compared to some other types of gastro‐intestinal cancer), longer follow‐up time may be required to provide a full picture on the long‐term effect.

To the best of our knowledge, this is the first study from a European setting investigating the association between *H. pylori* eradication and colorectal cancer risk. Our results should be generalizable to other (North) European settings with similar prevalence of *H. pylori*, comparable eradication policies, and colorectal cancer incidence. The previous cohort study from Taiwan included only 615 adults, regularly followed up for 9 years, and did find a three times higher risk of colorectal adenomas among those with a persistent *H. pylori* infection, compared to those successfully eradicated [21]. The Hong Kong population‐based cohort study included 96,572 individuals with successful eradication (median follow‐up of 9.7 years), of whom 1417 (1.5%) developed colorectal cancer [42]. This is markedly higher than our 274 (0.34%) on 80,381 individuals—although they also excluded those who were diagnosed with cancer or died within the first year [22]. The Hong Kong group used very similar methods, and did show an overall SIR = 1.03, so similar risks to the background population; and a clear decreasing trend (SIR 1.14 1–5 years; SIR = 1.00 6–10 years; SIR = 0.85 ≥ 11 years), especially for colon cancer—while the SIRs were all below one for rectal cancer [26]. These results are in line with what would be expected assuming *H. pylori* effectively decreases colorectal cancer risk. Important for the comparison with our results are the differences in our exposure and outcome, and other risk factors. The prevalence of *H. pylori* was estimated to be approximately 16% among adults in Sweden; while it was estimated around 15% in Hong Kong (and 34% in Taiwan) [24]. Yet, the “test‐and‐treat” approach for *H. pylori* is recommended in Hong Kong for individuals at high risk for gastric cancer [49], while *H. pylori* will in general only be detected in symptomatic patients in Sweden. The prevalence of *H. pylori* has been anyhow clearly declining both in Europe and South‐East Asia [50]. In Northern Europe, the colorectal cancer incidence is more than twice as high (33.6 per 100,000 person years) than in South‐East Asia (14.8 per 100,000 person years), which may suggest other risk factors for colorectal cancer may be more important in Sweden than *H, pylori*. Recognized modifiable risk factors include smoking, unhealthy diet (processed food/red meat), alcohol and obesity, while physical activity, nonsteroidal drugs and a healthy diet (high fiber intake), and menopausal hormone therapy appear to be protective [51, 52, 53]. Yet, there is also increasing evidence regarding environmental pollutants, and differences in exposure to risk factors in different birth cohorts [51]. This raises the question about critical windows regarding *H. pylori* exposure, and when eradication may come too late to prevent (colorectal) cancer and cancer progression‐ and how other (modifiable) risk factors, including prescribed drug use, may interact.

The observation that the SIRs for right‐sided colorectal cancer were potentially higher than for left‐sided colorectal cancer may suggest detection bias is less of a concern as left‐sided colon cancer is easier to detect due to its clinical symptoms [54, 55], and might be explained by its closers anatomical proximity to the upper gastrointestinal tract.

## 5 Conclusion

In summary, this study showed that the risk of colorectal cancer was higher than in the background population immediately after *H. pylori* eradication, and that a potential decrease may be present up to 6 years after treatment in this Northern‐European setting.

## Author Contributions

All authors designed the study and approved the final version of the manuscript (Q.L., O.S.‐A., L.E., K.F., N.B.). Q.L. drafted the protocol which was revised by all other authors. Q.L. conducted the statistical analyses under the supervision of N.B., Q.L. wrote the first draft of the manuscript, and prepared tables and figures. O.S.‐A., L.E., K.F., and N.B. critically revised the results and manuscript. Q.L. and N.B. had complete access to all the data and codes in the study. N.B. was the guarantor of the study.

## Conflicts of Interest

The authors declare no conflicts of interest.

## Supporting information


Appendix S1.

## Data Availability

The data cannot be shared in public repositories, as they contain highly detailed clinical information and belong the National Board of Health and Welfare. The data can be obtained after the required approvals are obtained by contacting the corresponding author.

## References

[1] E. Morgan , M. Arnold , A. Gini , et al., “Global Burden of Colorectal Cancer in 2020 and 2040: Incidence and Mortality Estimates From GLOBOCAN,” Gut 72, no. 2 (2023): 338–344. 36604116 10.1136/gutjnl-2022-327736

[2] Y. Zuo , Z. Jing , M. Bie , C. Xu , X. Hao , and B. Wang , “Association Between *Helicobacter pylori* Infection and the Risk of Colorectal Cancer: A Systematic Review and Meta‐Analysis,” Medicine 99, no. 37 (2020): e21832. 32925719 10.1097/MD.0000000000021832 PMC7489651

[3] D. S. Choi , S. I. Seo , W. G. Shin , and C. H. Park , “Risk for Colorectal Neoplasia in Patients With *Helicobacter pylori* Infection: A Systematic Review and Meta‐Analysis,” Clinical and Translational Gastroenterology 11, no. 2 (2020): e00127. 32032128 10.14309/ctg.0000000000000127 PMC7145030

[4] J. L. Wang , X. Liang , J. Xu , Y. X. Chen , and J. Y. Fang , “ *Helicobacter pylori* Infection Increases the Risk of Colorectal Adenomas: An Updated Meta‐Analysis,” Clinical Laboratory 64, no. 7 (2018): 1163–1170. 30146828 10.7754/Clin.Lab.2018.180115

[5] L. Ma , W. Guo , Z. Zeng , F. Yang , S. Tang , and Y. Ling , “Colorectal Cancer Risk in East Asian Patients With *Helicobacter pylori* Infection: A Systematic Review and Meta‐Analysis,” Medicine 102, no. 10 (2023): e33177. 36897722 10.1097/MD.0000000000033177 PMC9997759

[6] V. Engelsberger , M. Gerhard , and R. Mejías‐Luque , “Effects of *Helicobacter pylori* Infection on Intestinal Microbiota, Immunity and Colorectal Cancer Risk,” Frontiers in Cellular and Infection Microbiology 14 (2024): 1339750. 38343887 10.3389/fcimb.2024.1339750 PMC10853882

[7] A. K. Mohamed , N. M. Elhassan , Z. A. Awhag , et al., “Prevalence of *Helicobacter pylori* Among Sudanese Patients Diagnosed With Colon Polyps and Colon Cancer Using Immunohistochemistry Technique,” BMC Research Notes 13, no. 1 (2020): 322. 32631443 10.1186/s13104-020-05159-2 PMC7339555

[8] A. Soylu , S. Ozkara , H. Alis , et al., “Immunohistochemical Testing for *Helicobacter pylori* Existence in Neoplasms of the Colon,” BMC Gastroenterology 8 (2008): 35. 18702825 10.1186/1471-230X-8-35 PMC2527302

[9] Y. Wu , L. Shi , Q. Li , et al., “Microbiota Diversity in Human Colorectal Cancer Tissues Is Associated With Clinicopathological Features,” Nutrition and Cancer 71, no. 2 (2019): 214–222. 30843732 10.1080/01635581.2019.1578394

[10] C. C. Chen , J. M. Liou , Y. C. Lee , T. C. Hong , E. M. El‐Omar , and M. S. Wu , “The Interplay Between Helicobacter pylori and Gastrointestinal Microbiota,” Gut Microbes 13, no. 1 (2021): 1–22. 10.1080/19490976.2021.1909459 PMC8096336 33938378

[11] Z. Jiang , L. Li , J. Chen , et al., “Human Gut‐Microbiome Interplay: Analysis of Clinical Studies for the Emerging Roles of Diagnostic Microbiology in Inflammation, Oncogenesis and Cancer Management,” Infection, Genetics and Evolution 93 (2021): 104946. 10.1016/j.meegid.2021.104946 34052417

[12] M. Asghari‐Jafarabadi , S. Wilkins , J. P. Plazzer , R. Yap , and P. J. McMurrick , “Prognostic Factors and Survival Disparities in Right‐Sided Versus Left‐Sided Colon Cancer,” Scientific Reports 14, no. 1 (2024): 12306. 38811769 10.1038/s41598-024-63143-3 PMC11136990

[13] H. Ra , S. Jeong , H. Lee , et al., “Clinicopathological Differences Between Right and Left Colorectal Cancer by Sex,” Journal of Clinical Medicine 13, no. 10 (2024): 2810. 38792352 10.3390/jcm13102810 PMC11122515

[14] E. Doorakkers , J. Lagergren , L. Engstrand , and N. Brusselaers , “Eradication of *Helicobacter pylori* and Gastric Cancer: A Systematic Review and Meta‐Analysis of Cohort Studies,” Journal of the National Cancer Institute 108, no. 9 (2016): djw132. 27416750 10.1093/jnci/djw132

[15] F. Zhu , X. Zhang , P. Li , and Y. Zhu , “Effect of *Helicobacter pylori* Eradication on Gastric Precancerous Lesions: A Systematic Review and Meta‐Analysis,” Helicobacter 28, no. 6 (2023): e13013. 37602719 10.1111/hel.13013

[16] A. C. Ford , Y. Yuan , and P. Moayyedi , “Long‐Term Impact of *Helicobacter pylori* Eradication Therapy on Gastric Cancer Incidence and Mortality in Healthy Infected Individuals: A Meta‐Analysis Beyond 10 Years of Follow‐Up,” Gastroenterology 163, no. 3 (2022): 754–756. 35598628 10.1053/j.gastro.2022.05.027

[17] J. Butt and M. Epplein , “Helicobacter Pylori and Colorectal Cancer‐A Bacterium Going Abroad?,” PLoS Pathogens 15, no. 8 (2019): e1007861. 31393968 10.1371/journal.ppat.1007861 PMC6687094

[18] E. Doorakkers , J. Lagergren , V. K. Gajulapuri , S. Callens , L. Engstrand , and N. Brusselaers , “ *Helicobacter pylori* Eradication in the Swedish Population,” Scandinavian Journal of Gastroenterology 52, no. 6–7 (2017): 678–685. 28323552 10.1080/00365521.2017.1303844

[19] E. Doorakkers , J. Lagergren , L. Engstrand , and N. Brusselaers , “ *Helicobacter pylori* Eradication Treatment and the Risk of Gastric Adenocarcinoma in a Western Population,” Gut 67, no. 12 (2018): 2092–2096. 29382776 10.1136/gutjnl-2017-315363

[20] E. Doorakkers , J. Lagergren , G. Santoni , L. Engstrand , and N. Brusselaers , “ *Helicobacter pylori* Eradication Treatment and the Risk of Barrett's Esophagus and Esophageal Adenocarcinoma,” Helicobacter 25, no. 3 (2020): e12688. 32175626 10.1111/hel.12688

[21] K. C. Hu , M. S. Wu , C. H. Chu , et al., “Decreased Colorectal Adenoma Risk After *Helicobacter pylori* Eradication: A Retrospective Cohort Study,” Clinical Infectious Diseases 68, no. 12 (2019): 2105–2113. 30566695 10.1093/cid/ciy591

[22] C. G. Guo , F. Zhang , F. Jiang , et al., “Long‐term Effect of *Helicobacter pylori* Eradication on Colorectal Cancer Incidences,” Therapeutic Advances in Gastroenterology 16 (2023): 17562848231170943. 37168403 10.1177/17562848231170943 PMC10164860

[23] T. Grantham , R. Ramachandran , S. Parvataneni , D. Budh , S. Gollapalli , and V. Gaduputi , “Epidemiology of Gastric Cancer: Global Trends, Risk Factors and Premalignant Conditions,” Journal of Community Hospital Internal Medicine Perspectives 13, no. 6 (2023): 100–106. 38596548 10.55729/2000-9666.1252 PMC11000854

[24] Y. C. Chen , P. Malfertheiner , H. T. Yu , et al., “Global Prevalence of *Helicobacter pylori* Infection and Incidence of Gastric Cancer Between 1980 and 2022,” Gastroenterology 166, no. 4 (2024): 605–619. 38176660 10.1053/j.gastro.2023.12.022

[25] Y. Li , A. I. Hahn , M. Laszkowska , F. Jiang , A. G. Zauber , and W. K. Leung , “Global Burden of Young‐Onset Gastric Cancer: A Systematic Trend Analysis of the Global Burden of Disease Study 2019,” Gastric Cancer 27 (2024): 684–700. 38570392 10.1007/s10120-024-01494-6 PMC11193827

[26] I. O. Sawaid and A. O. Samson , “Proton Pump Inhibitors and Cancer Risk: A Comprehensive Review of Epidemiological and Mechanistic Evidence,” Journal of Clinical Medicine 13, no. 7 (2024): 1970. 38610738 10.3390/jcm13071970 PMC11012754

[27] T. H. Tran , S. K. Myung , and T. T. K. Trinh , “Proton Pump Inhibitors and Risk of Gastrointestinal Cancer: A Meta‑Analysis of Cohort Studies,” Oncology Letters 27, no. 1 (2024): 28. 38073768 10.3892/ol.2023.14161 PMC10698841

[28] K. Liu , Y. H. Wang , J. Wang , B. Chen , N. Luo , and J. Gong , “Meta‐Analysis of Proton Pump Inhibitor Use and the Risk Of Developing Gastric Cancer or Colorectal Cancer,” Anti‐Cancer Drugs 34, no. 9 (2023): 971–978. 37578746 10.1097/CAD.0000000000001418

[29] J. Simin , R. Fornes , Q. Liu , et al., “Antibiotic Use and Risk of Colorectal Cancer: A Systematic Review and Dose‐Response Meta‐Analysis,” British Journal of Cancer 123, no. 12 (2020): 1825–1832. 32968205 10.1038/s41416-020-01082-2 PMC7722751

[30] G. Qu , C. Sun , M. Sharma , et al., “Is Antibiotics Use Really Associated With Increased Risk of Colorectal Cancer? An Updated Systematic Review and Meta‐Analysis of Observational Studies,” International Journal of Colorectal Disease 35, no. 8 (2020): 1397–1412. 32504337 10.1007/s00384-020-03658-z

[31] S. S. M. Lu , Z. Mohammed , C. Häggström , et al., “Antibiotics Use and Subsequent Risk of Colorectal Cancer: A Swedish Nationwide Population‐Based Study,” Journal of the National Cancer Institute 114, no. 1 (2022): 38–46. 34467395 10.1093/jnci/djab125 PMC8755503

[32] S. M. Wallerstedt , B. Wettermark , and M. Hoffmann , “The First Decade With the Swedish Prescribed Drug Register—A Systematic Review of the Output in the Scientific Literature,” Basic & Clinical Pharmacology & Toxicology 119, no. 5 (2016): 464–469. 27112967 10.1111/bcpt.12613

[33] E. von Elm , D. G. Altman , M. Egger , S. J. Pocock , P. C. Gøtzsche , and J. P. Vandenbroucke , “The Strengthening the Reporting of Observational Studies in Epidemiology (STROBE) Statement: Guidelines for Reporting Observational Studies,” Lancet 370, no. 9596 (2007): 1453–1457. 18064739 10.1016/S0140-6736(07)61602-X

[34] M. Zamani , S. Alizadeh‐Tabari , V. Zamani , J. Shokri‐Shirvani , and M. H. Derakhshan , “Worldwide and Regional Efficacy Estimates of First‐Line *Helicobacter pylori* Treatments: A Systematic Review and Network Meta‐Analysis,” Journal of Clinical Gastroenterology 56, no. 2 (2022): 114–124. 34855643 10.1097/MCG.0000000000001641

[35] S. G. Thrumurthy , S. S. Thrumurthy , C. E. Gilbert , P. Ross , and A. Haji , “Colorectal Adenocarcinoma: Risks, Prevention and Diagnosis,” BMJ 354 (2016): i3590. 27418368 10.1136/bmj.i3590

[36] J. D. Vogel , S. I. Felder , A. R. Bhama , et al., “The American Society of Colon and Rectal Surgeons Clinical Practice Guidelines for the Management of Colon Cancer,” Diseases of the Colon and Rectum 65, no. 2 (2022): 148–177. 34775402 10.1097/DCR.0000000000002323

[37] N. E. Breslow and N. E. Day , “Statistical Methods in Cancer Research,” in Vol II Design and Analysis of Cohort Studies International Agency for Research on Cancer (Lyon: WHO, 1987), 82–118. 3329634

[38] G. Sandblom , M. Dufmats , M. Olsson , and E. Varenhorst , “Validity of a Population‐Based Cancer Register in Sweden an Assessment of Data Reproducibility in the South‐East Region Prostate Cancer Register,” Scandinavian Journal of Urology and Nephrology 37, no. 2 (2003): 112–119. 12745718 10.1080/00365590310008839

[39] B. Wettermark , N. Hammar , C. M. Fored , et al., “The New Swedish Prescribed Drug Register—Opportunities for Pharmacoepidemiological Research and Experience From the First Six Months,” Pharmacoepidemiology and Drug Safety 16, no. 7 (2007): 726–735. 16897791 10.1002/pds.1294

[40] N. Brusselaers , A. Vall , F. Mattsson , and J. Lagergren , “Tumour Staging of Oesophageal Cancer in the Swedish Cancer Registry: A Nationwide Validation Study,” Acta Oncologica 54, no. 6 (2015): 903–908. 25800722 10.3109/0284186X.2015.1020968

[41] L. Barlow , K. Westergren , L. Holmberg , and M. Talback , “The Completeness of the Swedish Cancer Register: A Sample Survey for Year 1998,” Acta Oncologica 48, no. 1 (2009): 27–33. 18767000 10.1080/02841860802247664

[42] N. Brusselaers , K. Wahlin , L. Engstrand , and J. Lagergren , “Maintenance Therapy With Proton Pump Inhibitors and Risk of Gastric Cancer: A Nationwide Population‐Based Cohort Study in Sweden,” BMJ Open 7, no. 10 (2017): e017739. 10.1136/bmjopen-2017-017739 PMC5665226 29084798

[43] E. Altobelli , A. Lattanzi , R. Paduano , G. Varassi , and F. di Orio , “Colorectal Cancer Prevention in Europe: Burden of Disease and Status of Screening Programs,” Preventive Medicine 62 (2014): 132–141. 24530610 10.1016/j.ypmed.2014.02.010

[44] H. Thorlacius and E. Toth , “Implementation of Colorectal Cancer Screening in Sweden,” Läkartidningen 115 (2018): E3PI. 29809270

[45] P. Moberger , F. Sköldberg , and H. Birgisson , “Evaluation of the Swedish Colorectal Cancer Registry: An Overview of Completeness, Timeliness, Comparability and Validity,” Acta Oncologica 57, no. 12 (2018): 1611–1621. 30477372 10.1080/0284186X.2018.1529425

[46] K. Murakami and H. Matsubara , “Chronology of Gastrointestinal Cancer,” Surgery Today 48, no. 4 (2018): 365–370. 28795270 10.1007/s00595-017-1574-y PMC5845061

[47] W. D. Chey , G. I. Leontiadis , C. W. Howden , and S. F. Moss , “ACG Clinical Guideline: Treatment of *Helicobacter pylori* Infection,” American Journal of Gastroenterology 112, no. 2 (2017): 212–239. 28071659 10.1038/ajg.2016.563

[48] S. C. Shah , M. C. Camargo , M. Lamm , et al., “Impact of *Helicobacter pylori* Infection and Treatment on Colorectal Cancer in a Large, Nationwide Cohort,” Journal of Clinical Oncology 42, no. 16 (2024): 1881–1889. 38427927 10.1200/JCO.23.00703 PMC11588569

[49] W. K. Leung , K. S. Cheung , P. C. O. Sham , et al., “Consensus Recommendations for the Screening, Diagnosis, and Management of Helicobacter Pylori Infection in Hong Kong,” Hong Kong Medical Journal 29, no. 6 (2023): 532–541. 37385947 10.12809/hkmj2210321

[50] Y. Li , H. Choi , K. Leung , F. Jiang , D. Y. Graham , and W. K. Leung , “Global Prevalence of *Helicobacter pylori* Infection Between 1980 and 2022: A Systematic Review and Meta‐Analysis,” Lancet Gastroenterology & Hepatology 8, no. 6 (2023): 553–564. 37086739 10.1016/S2468-1253(23)00070-5

[51] C. C. Murphy and T. A. Zaki , “Changing Epidemiology of Colorectal Cancer—Birth Cohort Effects and Emerging Risk Factors,” Nature Reviews. Gastroenterology & Hepatology 21, no. 1 (2024): 25–34. 37723270 10.1038/s41575-023-00841-9

[52] Y. Liu , C. Zhang , Q. Wang , et al., “Temporal Trends in the Disease Burden of Colorectal Cancer With Its Risk Factors at the Global and National Level From 1990 to 2019, and Projections Until 2044,” Clinical Epidemiology 15 (2023): 55–71. 36659904 10.2147/CLEP.S388323 PMC9842526

[53] Y. Liang , N. Zhang , M. Wang , et al., “Distributions and Trends of the Global Burden of Colorectal Cancer Attributable to Dietary Risk Factors Over the Past 30 Years,” Nutrients 16, no. 1 (2023): 132. 38201962 10.3390/nu16010132 PMC10780867

[54] B. Baran , N. Mert Ozupek , N. Yerli Tetik , E. Acar , O. Bekcioglu , and Y. Baskin , “Difference Between Left‐Sided and Right‐Sided Colorectal Cancer: A Focused Review of Literature,” Gastroenterology Research 11, no. 4 (2018): 264–273. 30116425 10.14740/gr1062w PMC6089587

[55] M. Mik , M. Berut , L. Dziki , R. Trzcinski , and A. Dziki , “Right‐ and Left‐Sided Colon Cancer—Clinical and Pathological Differences of the Disease Entity in One Organ,” Archives of Medical Science 13, no. 1 (2017): 157–162. 28144267 10.5114/aoms.2016.58596 PMC5206358

