# Supplementary material for: Helicobacter pylori Eradication Therapy and the Risk of Colorectal Cancer: A Population‐Based Nationwide Cohort Study in Sweden
Source: Helicobacter. 2024 Nov 20;29(6):e70001. doi: 10.1111/hel.70001 (PMC11578941; doi:10.1111/hel.70001)
Supplement: Supplementary file 1 — Appendix S1. [file HEL-29-e70001-s001.docx]

Supplementary Table S1. Codes for international classification of diseases, 10^th^ edition (ICD-10) and anatomical therapeutic chemicals (ATC)

| Diseases | ICD-10 codes | Dispensed drugs | ATC codes |
| --- | --- | --- | --- |
| Colon cancer | C18 | *Helicobacter pylori* eradication recommended regimen | Package (A02BD06), and one proton pumps inhibitor (A02BC) with two of the antibiotics (amoxicillin J01CA04, clarithromycin J01FA09, or metronidazole J01XD01) |
| Adenocarcinoma | 096 |  |  |
| Right-sided colon cancer | C18.0 C18.1 C18.2 C18.3 |  |  |
| Transverse colon cancer | C18.4 |  |  |
| Unspecified sites of colon cancer | C18.8, C18.9 |  |  |
| Left-sided colon cancer | C18.5 C18.6 |  |  |
| Sigmoid colon cancer | C18.7 |  |  |
| Rectum cancer* | C19 C 20 |  |  |
| Anal cancer | C21 |  |  |

*In this study, count rectum cancer = rectosigmoid border cancer + rectal cancer

Supplementary Figure S1. Flowchart of exclusion criteria, with individual counts.

140,391 unique individuals

Exclude people who took the first eradication after Jan 2012 (N =26,717 individuals)

113,674 unique individuals

Exclude age at first prescription < 18 (N =3,513 individuals)

110,161 unique individuals

Exclude people took alternative regimens (N =4,540 individuals)

105,621 unique individuals

Exclude people diagnosed as any cancer (except non-skin melanoma) before Jul2005 & before last eradication (N =8,203 individuals)

97,418 unique individuals

Exclude people with <1 year follow-up from the last eradication episode (N = 17,037 individuals)

80,381 unique individuals
